# Easily-Deployable Acoustic Local Positioning System Based on Auto-Calibrated Wireless Beacons

**DOI:** 10.3390/s19061385

**Published:** 2019-03-20

**Authors:** José A. Moreno, Fernando J. Álvarez, Teodoro Aguilera, José A. Paredes

**Affiliations:** Sensory System Research Group, University of Extremadura, 06006 Badajoz, Spain; fafranco@unex.es (F.J.Á.); teoaguibe@unex.es (T.A.); japaredesm@unex.es (J.A.P.)

**Keywords:** Acoustic Local Positioning System (ALPS), wireless beacons, auto-calibration, digital signal processing, embedded systems

## Abstract

Self-calibrated Acoustic Local Positioning Systems (ALPS) generally require a high consumption of hardware and software resources to obtain the user’s position at an acceptable update rate. To address this limitation, this work proposes a self-calibrated ALPS based on a software/hardware co-design approach. This working architecture allows for efficient communications, signal processing tasks, and the running of the positioning algorithm on low-cost devices. This fact also enables the real-time system operation. The proposed system is composed of a minimum of four RF-synchronized active acoustic beacons, which emit spread-spectrum modulated signals to position an unlimited number of receiver nodes. Each receiver node estimates the beacons’ position by means of an auto-calibration process and then computes its own position by means of a 3D multilateration algorithm. A set of experimental tests has been carried out where the feasibility of the proposed system is demonstrated. In these experiments, accuracies below 0.1 m are obtained in the determination of the receptor node position with respect to the set of previously-calibrated beacons.

## 1. Introduction

The applications and services of people and object localization and context analysis are a growing demand in consumer society, so it is necessary to investigate and develop alternative and complementary technologies to existing global location [1] to cover also indoor environments with better performance, precision, and robustness [2,3].

Most indoor positioning systems proposed so far can be classified into three main categories. First are those based on the measurement of radio frequency Received Signal Strength (RSS). These systems require an external infrastructure to generate the RF signals, but they usually benefit from already deployed WPAN [4,5] or WLAN [6,7] transceivers. This technology provides a positioning accuracy between one and a few meters, due to the complex propagation mechanisms of RF signals indoors that cause a large variability of their RSS measurements. Secondly, we find those systems that make use of inertial sensors (accelerometers and gyroscopes) to perform dead reckoning [8,9]. These systems do not need an external infrastructure to operate, but they provide low positioning accuracy due to their inherent cumulative error, typically around 10% of the total traveled distance. Finally, the third category covers all those systems that measure either the Time of Arrival (ToA) or the Time Difference of Arrival (TDoA) of the acoustic signals emitted from a set of fixed beacons, to render the target’s location by following a trilateration or multilateration procedure, respectively [10]. By using encoded emissions and pulse compression techniques to detect them, these systems can achieve centimetric positioning accuracy, high robustness to in-band noise, and multiple access capability [11].

Nevertheless, one of the main inconveniences that are attributed to Acoustic Local Positioning Systems (ALPS) is their limited scalability. The only way to extend the operational area of these systems is to deploy new beacons at known and precise locations, whose coordinates will be used to solve the trilateration or multilateration equations, provided that the simultaneous coverage of a minimum of three or four beacons is always ensured, respectively. Needless to say, this is a tedious task prone to multiple errors during the beacons’ placement procedure, even more given the usually wired infrastructure of these beacons.

To overcome the problem stated above, this work proposes a novel ALPS based on a set of easily-deployable wireless beacons, whose main feature is their capability to conduct an auto-calibration process that eliminates the need to measure their precise location during deployment. Several works can be found in the literature that have already proposed the auto-calibration of a set of acoustic beacons. Hence, in [12], the authors proposes a Simultaneous Calibration and Navigation (SCAN) algorithm for Mobile Robots (MR) over a wide area. This system consists of a set of ALPS, some of which are calibrated and Globally Referenced (GRALPS), whereas the rest are not calibrated and Locally Referenced (LRALPS). The MR is in charge of calibrating the beacons of the different LRALPS under which it moves by using multiple filters running in parallel (as many as GRALPS and LRALPS). This allows obtaining the transformation vector that converts the coordinates of the local reference system to the global system. The precision obtained in the estimation of the beacons’ location is below 0.2 m and 0.5 m when trilateration or multilateration are used, respectively. In [13], a self-calibration algorithm was also proposed to calculate the position of a set of ultrasonic transmitters using the nonlinear least squares minimization principle and a set of distance readings previously obtained by a walking user. This method achieves an accuracy of 20 cm in the determination of a set of four beacons’ location. However, it is also worth mentioning that this system stops working when the beacons are arranged in the same plane or describing a circle. Furthermore, in [14], the self-calibration of an ultrasonic location system was implemented using two methods. The first method (explorer) used three pre-calibrated beacons to explore the complete positioning area using the Time Division Multiple Access (TDMA) protocol, while the second method (distribute and erase) progressively refined the position of the remaining beacons using an adapted trilateration system. By combining these methods, it is possible to reach accuracies of 1 mm with a distribution of 11 × 11 beacons and after 1200 algorithm iterations. Nevertheless, the main limitation of this system resides in the small exploration area where it operates. Finally, in [15], the authors proposed the LOSNUS system (Localization of Sensor Nodes by Ultrasound) based on the measurement of the ToFs of a set of six transmitters and four receivers that together with the speed of sound allow defining a system of 24 equations that, once solved through a minimization algorithm, allows the definition of a system of coordinates. Using this method, calibration accuracies of less than one centimeter were achieved.

A key feature of the system proposed in this work is its implementation through hardware/software co-design [16]. This architecture of operation allows: (1) the integration of RF synchronization devices and acoustic sensors in low-cost System on Chip (SoC) platforms and (2) the efficient running of signal processing tasks and a positioning algorithm that enable real-time system operation.

The remainder of the manuscript is organized as follows: Section 2 gives a brief system overview; Section 3 describes the proposed emitter subsystem (beacons) and signals’ generation; Section 4 explains in detail the estimation of the position and the receiver subsystem; Section 5 presents the practical implementation and some results; finally, the main conclusions of this work are given in Section 6.

## 2. General Overview

This section offers an overview of the entire system, as well as the signals in the acoustic communication. The system has been built focusing on both the emission and the reception of the acoustic signals mentioned in Section 1. Besides, it is supported by similar platforms based on the ARM [17] Cortex-M4 [18] SoC architectures. Their main characteristics will be detailed in later sections.

**Figure 1:**
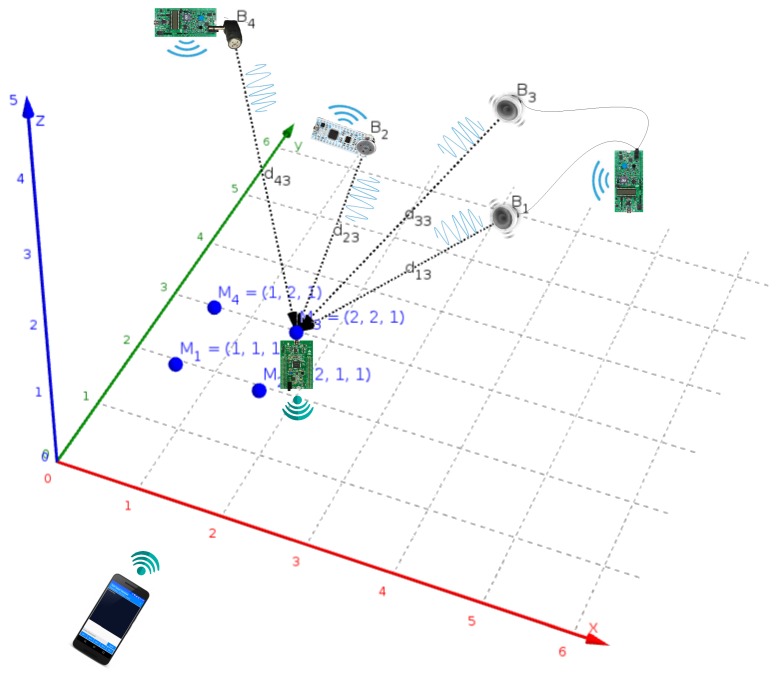
Overview of the proposed local positioning system.

In Figure 1, the four beacon nodes are depicted as $B_{i}$ with i∈{1,2,3,4}. It is possible to configure the system with different implementations, but the choice has been made with the aim of minimizing the cost. There are two operation modes: the most advanced alternative supports a dual beacon operation mode by using a set of home stereo speakers ($B_{1}$ and $B_{3}$). The simplest mode uses individual loudspeakers, soldered ($B_{2}$) or jack encapsulated ($B_{4}$), chosen due to their small size and good performance for the desired functionality. All nodes also communicate via an RF protocol that provides the required synchronization to conduct the simultaneous emission of acoustic signals.

Moreover, the 3D points $M_{j}$ with j∈{1,2,3,4} depict measured locations where the receiver platform, which includes a MEMS audio sensor, performs the signal acquisition and processing. The beacons are previously calibrated, and their results are remotely communicated from an adapted WiFi interface to a smartphone that shares a WLAN network.

## 3. Beacons’ Structure and Signals’ Generation

In order to implement a flexible and low-cost infrastructure of acoustic emission beacons, the use of energy-saving SoC-based devices has been considered. These devices can be powered by long-lasting batteries, communicate with each other wirelessly, and transmit, in a synchronized way between them, encoded sequences. This section describes the designed hardware platform, the coding of the sequences to be transmitted and the hardware/software implementation of the beacons system, including the synchronization between them.

**Figure 2:**
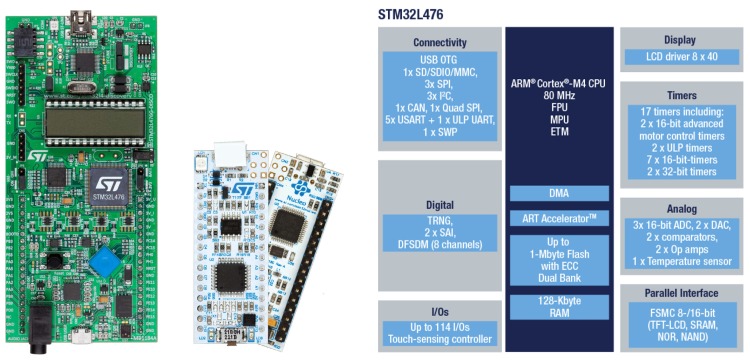
Development kits L476Discovery (**left**), L432Nucleo (**middle**), and a summary of resources in STM32 SoC included (**right**).

A development board based on the 32-bit micro-controller ARM Cortex-M4 has been used for beacon prototyping. This board requires minimum performance and storage, and it is able to carry out DAC conversion. It also has some programmable timers and clock sources. This prototype works at a low power consumption rate, and it features an efficient power management capability. Each beacon makes use of an independent battery. Besides, the L4 [19] family of MCUs from ST Microelectronics [20] has been chosen, which stands out in performance and Ultra-Low Power (ULP) consumption. The available set of interfaces and peripherals in SoC STM32L476 [21] of Discovery Kit 32L476 GDISCOVERY [22] (Figure 2 left) from ST is represented on the right-hand side of the figure, although for the corresponding application, we will make use of 2× DAC, 2× SPI, 1× USART, 1× channel, 1× timer, internal SRAM, and Flash memory for the storage of generated signal samples and the LCD controller and GPIO inputs of joystick cursors for the minimal user interface.

External to SoC, some PCB peripherals were used. Noteworthy are the audio stereo DAC CS43L22 with a Class D amplifier for external mono/stereo speakers via the jack connector, several GPIO (General Purpose Input/Output) pins, like LEDs, push buttons, joystick, and an LCD for the user interface. The SAI module in combination with the audio DAC is essential in the beacon’s application, as an alternative to the SoC DAC, in order to support a dual beacon by stereo DAC code outputs. Moreover, a rear socket enables supply to the rest of the components in the base PCB through a 3.0-V lithium manganese battery with a nominal capacity of 200 mAh.

**Figure 3:**
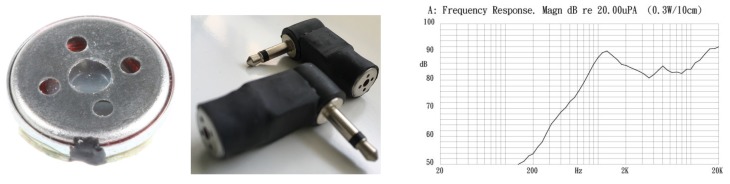
Miniature speaker KDMG10008C encapsulated with jack connectors and its frequency response.

In order to enable wireless communication between the beacons and to carry out the necessary synchronization for the simultaneous emission of codes, the module MRF89XAM8A [23] of Microchip [24] has been added (Figure 3). This module is a multichannel FSK/OOK transceiver operating in the 863–870 MHz ISM frequency band. It has a PCB antenna, based on the Microchip MRF89XA [25] single-chip RF transceiver. The main characteristics are the sensitivity of −107 dBm to 25 Kbps FSK with digital RSSI, the current consumption of 25 mA in transmission and 3 mA in reception, with modes of low consumption in periods of inactivity, an FIFO of 64-byte for transmit and receive data, and recognition of input synchronized words (patterns). The connection with the host platform is made by means of SPI facilities.

Finally, a miniature magnetic speaker (10 mm) of 0.3 W and 8Ω by Kingstate has been used with the previous amplifier as part of the ALPS acoustic subsystem (Figure 3). As can be seen in the middle image of this figure, an alternative assembly has been made with the audio mono jack connection via SAI to the Discovery platform.

**Figure 4:**
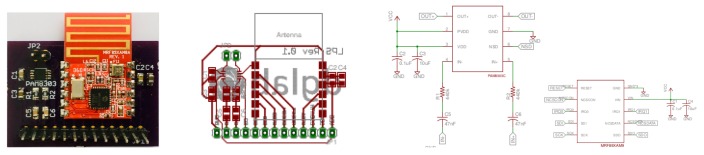
PCB views of the ALPS beacon including the MRF89XAM8A RF 868-MHz transceiver module.

Each beacon has been modeled around a designed PCB (Figure 4), connected to a development platform to emit pattern codes generated from binary sequences, whose modulation symbol has been stored in their RAM blocks.

This set of components forms each beacon, which operates simultaneously with the rest of the nearby beacons by making use of the wireless connection provided by the RF transceiver module. After synchronization, these beacons emit their corresponding patterns, previously programmed through an USB port from a computer. This system is powered by its own battery incorporated in a socket of the MCU kit.

### 3.1. Issued Sequences

The sequences’ emission is carried out with the HW structure by means of the corresponding execution of the FW programmed in the flash memory of the MCU.

In the description of the developed SW and its operation, two different parts can be distinguished. They are, in sequential order, (a) the synchronization stage between environment beacons through the RF transceiver, which it is carried out in a timely way at the beginning of the system (it also serves to resynchronize possible temporary drifts); and (b) the generation of periodic sequences by means of the DAC and the acoustic transducer.

To enable TOF-based positioning, all acoustic beacons fire their emissions simultaneously. The choice of the codes has been based on getting low cross-correlation values to reduce the effects of multiple access interference. Several families of sequences have been studied for these systems, for instance, gold codes, Complementary Sequence Sets (CSS), and LS (Loosely Synchronized) codes. However, in a recent work [26], it has been demonstrated that not all of these families are equally resilient to adverse effects such as multi-access, multipath, and Doppler shift. Kasami sequences are known to represent a good compromise between the good cross-correlation values and the slow degradation of these properties associated with the movement of the receiver. Kasami sequences belong to the well-known group of pseudo-random sequences (PN), which can be generated using Linear Feedback Shift Registers (LFSR) and XOR logic gates [27]. A new Kasami sequence can be obtained from an *m* sequence and the decimated and chained version of this sequence carrying out the module two addition between the first and the delayed version of the second one, that is:

(1) $$c=m_{1}⊕D^{l}m_{2}\;\mathrm{with}\;l<L$$

where $m_{1}$ is the maximal sequence of length $L=2^{N}-1$ with *N* even; $m_{2}$ is the obtained sequence decimating $m_{1}$ a factor of $q=2^{N/2}+1$ and concatenating result *q* times; ⊕ is the module two sum operator and $D^{l}m_{2}$ the resulting sequence by circular shifting *l* positions the $m_{2}$ sequence. Following this procedure, families of Kasami sequences have been generated for N=6,8,10, with L=63,255,1023 bits, respectively, each, from which the set of four sequences with better values has extracted lower cross-correlation as emission patterns for the LPS in each case.

As is well known, the higher the sequence length, the better the correlation under noisy critical conditions, so increasing this size can lead to a greater reliability and robustness of the system. As a counterpart, the larger the emission period, the heavier the computational load at the receiver.

**Figure 5:**
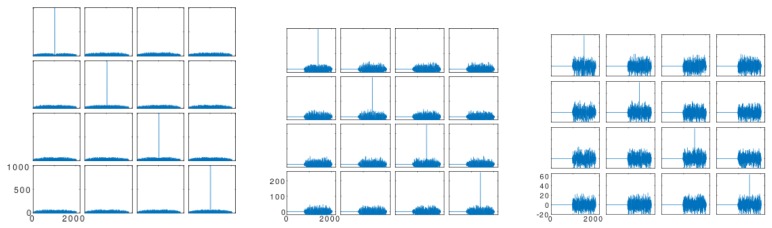
Autocorrelation of generated Kasami codes with 1023 bits (**left**), 255 bits (**middle**), and 63 bits (**right**).

In the proposed system, sequences of (1023) bits (Figure 5 left) were used in the following way: Four codes of 255 bits have been generated from four Kasami codes of 1023 bits with better properties (Figure 5 middle) by taking the central part of the previous ones (bits from 512−127–512+127). Similarly, four codes of 63 bits were generated by taking the bits from 512−31–512+31 (Figure 5 on the right). All families thus generated retain good cross-correlation properties, quasi-optimal orthogonal performance, and good cross-correlation values with the parent codes of the 1023-bit family.

The four sequences have been digitally modulated with a Binary Phase Shift Modulation (BPSK) scheme, allowing the adaptation of the emission spectrum to the frequency response of the acoustic transducers. This modulation scheme has been widely used to transmit binary codes in sonar systems, performing the detection of received echoes by pulse compression. Each bit of code c[n] is modulated in the proposed BPSK scheme with only one period of carrier signal, whose phase, zero or π, is given by the value of the corresponding bit to obtain the modulated pattern as:

(2) $$p\left[n\right]=\sum_{i=0}^{L-1}c\left[i\right]\cdot s\left[n-i\cdot M\right]$$

where *L* is the code length, s[n] represents the modulation symbol, and *M* is the number of samples in this symbol, whose value is given by the product between the number of carrier cycles that constitute the symbol and the quotient between the sampling and carrier frequencies.

### 3.2. Implementation

**Figure 6:**
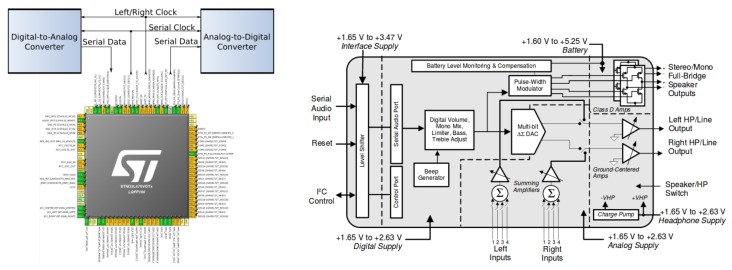
Simplified scheme of the SAI interface with the SoC pinout and the functional diagram of audio DAC.

**Figure 7:**
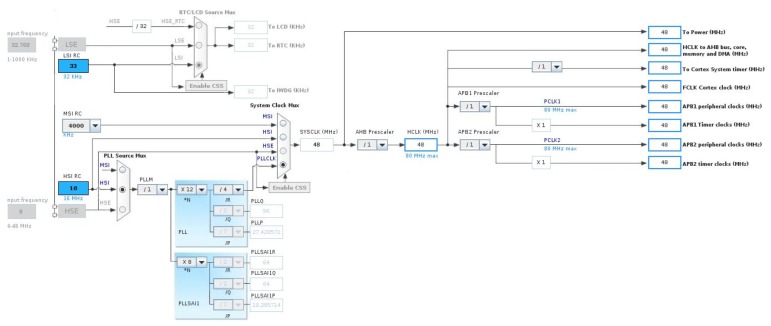
Clock settings for signal generation with STM32L476VG.

The resources used in the MCU for this phase were: a timer, a DAC, and a DMA channel between the memory and the peripherals. The pin configuration was required to use external resources such as the DAC output, as shown in Figure 6, in addition to a correct clock configuration for the use of the timer and other resources of the MCU (Figure 7).

The DAC module in the STM32 was a 12-bit converter with two output channels that support audio functions. Only one of these channels will be necessary in the proposed application. The data format used was 12 bits aligned to the right, giving a range of $000_{16}$–${\mathrm{FFF}}_{16}\left(4096_{10}\right)$. To start the conversion of the DAC channel, there were timers associated with a trigger register. In this case, the conversion started with the update event of a timer, which corresponded to its overflow. The timer was configured with a sampling frequency of $f_{s}$ = 96 kHz. The system clock was set as a multiple of that frequency to favor the exact division and precisely obtain $f_{s}$ in the timer.

In order to handle external loads without using an operational amplifier, the DAC channels integrate output buffers that can be activated depending on the application, thus avoiding any voltage drop with respect to the reference voltage. The use of this functionality was necessary in our case to obtain a maximum output voltage level of $V_{ref}$ = 3.3 V. If the digital value of the samples was in the range [−1,1], it needed to be calibrated again to have a positive value between zero and ${\mathrm{FFF}}_{16}$ (corresponding to the range from 0 V–3.3 V):

(3) $$y_{digital}\left(x\right)=\left(s\left(x\right)+1\right)\frac{\left(0xFFF+1\right)}{2}$$

The digital outputs were converted to output voltages by a linear conversion between zero and $V_{ref}+$, being determined by the following expression:

(4) $$DAC_{out}=y_{analog}\left(x\right)=V_{ref}\frac{y_{digital}\left(x\right)}{DAC_{maxval}}=3.3V\frac{y_{digital}\left(x\right)}{\left(0xFFF+1\right)}$$

The sample value table was saved in memory and transferred by DMA, in steps triggered by the same timer as the DAC.

In addition, two Serial Audio Interfaces (SAI) are available on the STM32 for digital audio devices’ connection. This interface is one of the most common mechanisms for transferring data from two audio channels between devices within a system, selecting between several standard protocols such as $I^{2}S$ [28], PCM-coded pulse modulation, Intel AC’97 [29], or user-defined ones. As can be seen in Figure 6, in brief, a SAI can establish a communication bus with ADC and DAC. One of the SAI interfaces of the STM32 was used in this case to communicate with the audio DAC CS43L22 [30] on the platform STM32L476 via bus $I^{2}C$ in address $94_{16}\left(148_{10}\right)$ and by means of a stereo jack connector to an omnidirectional MEMS microphone, which provides a Pulse Density Modulated (PDM) digital signal.

The alternative of using SAI with audio DAC from the CS43L22 codec with respect to the initial DAC configuration has several advantages. On the one side, it simplifies the PCB design without a Class D amplifier already included in the codec; on the other side, data scaling becomes unnecessary for the unsigned format with 12 bit with respect to inherent the flexibility of data types and configuration in codec via registers and BSPs from the STM32.

The transfer of the 1023-bit code, BPSK modulated with an oversampling factor ($\frac{f_{s}}{f_{c}}=6$), was carried out by associating a buffer with 6138 samples to a DMA channel connected to the SAI, making possible a direct transmission from memory without the need for a CPU intervention. The periodicity of the emission was obtained through a circular configuration of the pointed DMA buffer.

### 3.3. Inter-Beacons’ Synchronization

The SoC resources used for this phase consisted solely of the SPI peripheral for serial communication with the RF module, although the LCD mini-display and GPIO pins at the BSP level were also used for debugging purposes. The pin-out configuration is shown in Figure 6 and the clock configuration for these resources in Figure 7.

In this case, the SW development was based on the construction of an RF module device driver for the basic transmission and reception initialization functions. From these functions, a simple protocol was used by a beacon with master functions to send a synchronization message to the rest of the slave beacons. The slave ones responded with a configuration message and restarted the corresponding sequence-sending timer.

## 4. Receiver Module and Signal Processing

**Figure 8:**
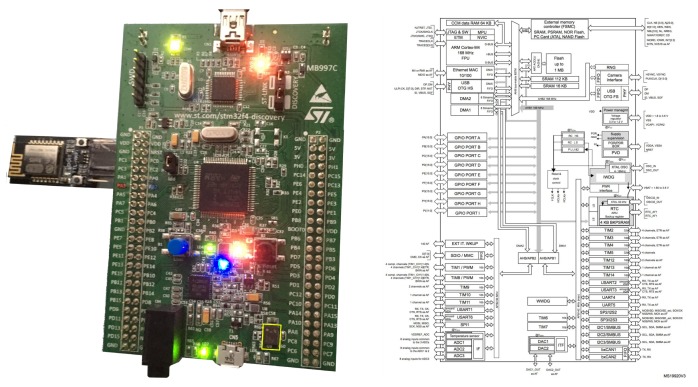
STM32F4Discovery board with MP45DT02 and STM32F407VG SoC overview.

**Figure 9:**
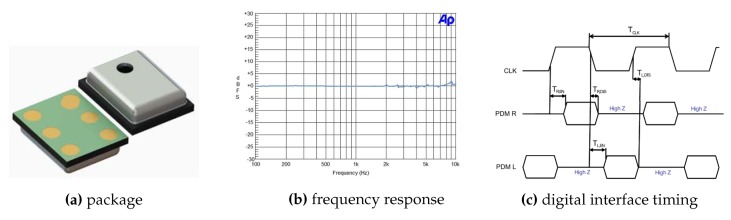
MP45DT02 MEMS audio sensor.

The receiver prototype was based on the evaluation board STM32F4 Discovery Kit [31,32] (Figure 8) assembled with the 32-bit microcontroller ARM [17] Cortex-M4 [18] STM32F407VG [33] from the F4 [34] family of ST Microelectronics [20] MCUs, suitable for RT audio processing. Its main resources are 168-MHz CPU, FPU, and ART accelerator, equipped with the MEMS audio sensor omnidirectional digital microphone MP45DT02 [35] (Figure 9), along with a CS43L22 DAC [30]. Similar to the rest of the SoC family, it includes multiple interfaces and peripheral resources, as shown on the right-hand side of Figure 8.

As depicted in this figure, a serial WiFi module (DT-06 Wireless WiFi) [36] was attached via an UART interface of the main board to interact remotely during the calibration process and to obtain positioning data from a smartphone in wireless mode.

### 4.1. MEMS Microphone

Digital MEMS audio sensors are quite inexpensive to make, apart from the obvious advantage of its tiny size. They also provide better isolation from nearby RF signals (as from a cell phone transmitter) and simplify the electronic design by reducing the required supporting circuits to just PCB traces for clock and data, power supply, and a configuration pin.

The MEMS microphone used in this system was the MP45DT02 [35]. Its main characteristics are the reduced size (HLGA package: 4.72 × 3.76 × 1.25 mm), the low power consumption, and the omnidirectional sensing. The aperture to capture sound is approximately 1 mm in diameter. If the stages most prone to noise effects are under control, well shielded, and isolated from the rest of the board system, the digital audio stream can be of higher quality (61 dB SNR, −26 dBFS sensitivity, 120 dBSPL AOP).

In order to deal with the received audio signal, there is a high bit rate serial signal input (1–3.25 MHz) for two channels (timings on Figure 9c). It requires digital signal processing to transform the audio signal into the data standard for targeted audio application handled by the micro-controller, in this case for positioning purposes. The format in which peripherals deliver data is Pulse Density Modulation (PDM) [37].

### 4.2. From PDM to PCM

Creating a PDM bit stream from the analog measurement of the membrane’s position is a well-understood design problem that is closely related to a class of analog-to-digital converters called sigma delta converters.

A smaller and reliable serial connection was set up in this system, since a 12- or 16-bit precision would require many parallel wires, pins, or even complicated serial protocols. STM32 microcontrollers offer Serial Peripheral Interface (SPI) blocks to communicate with external devices. Some of them also have the possibility of using the Inter-IC Sound audio protocol (I2S), a well-established serial protocol for sound data that uses a data wire, a bit clock, and a frame clock to convey pairs of PCM samples. For a microphone, the I2S has a clear disadvantage: the signal quality depends on the algorithms inside the microphone.

Most digital MEMS microphones use PDM instead of PCM. The former provides a much higher sample rate at a lower precision, which moves most of the parts of the signal processing into the receiving device and out of the microphone’s package, while still providing a digital interface [38]. This has a direct effect on the sound quality.

PDM represents the diaphragm position, which is proportional to the instantaneous air pressure variation, in a binary way (one or zero). For example, a dead silent room will produce a stream of alternating ones and zeroes; a 1-kHz tone will appear as a first half of the ones and a second half of the zeroes, in repeating pattern (depending on sample rate: in this case, 2.8224 MHz).

The described MP45DT02 MEMS microphone expects to be clocked at any rate between 1 MHz and 3.25 MHz, which produces finished PCM audio samples at all the common PCM rates ranging from 16 kHz (1.024 MHz)–48 kHz (3.072 MHz) when down-sampled by a factor of 64.

**Figure 10:**
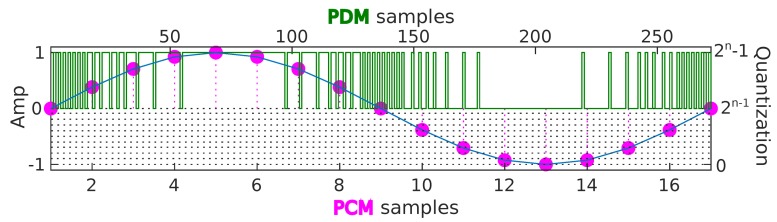
From analog signal to PDM and PCM processing.

Given the high data rate of the PDM bit stream, recovering a lower data rate PCM bit stream (Figure 10) is easily done by applying a suitable Low Pass Filter (LPF) with decimation, normally a factor of 64. The first stage of decimation is used to reduce the sampling frequency, followed by a High Pass Filter (HPF) to remove the signal DC offset.

Since the density of ones is proportional to the audio amplitude, the beginning can simply consist of taking a Moving average (MA) of the PDM samples. This is a basic form of a Finite Impulse Response (FIR) filter; however, it is still an average of 1-bit samples. In order to provide as much useful information as possible with each averaged sample, an average of a unique set of PDM bits is wanted. To achieve this, oversampling the PDM microphone by the same factor is needed.

So as to limit aliasing effects, the LPF is applied after the sampling process. LPFs can be implemented with an FIR filter. The LPF’s coefficients must be carefully chosen so that the filtering process reduces the effects of any high frequency components in the stream. It is worth noting that the number of taps in the FIR filter can be equal to or greater than the desired decimation factor. A good reason for a greater number of taps is to improve the filter attenuation of high frequencies. More taps will be required to produce more granular signal levels in the output signal. However, a higher PDM sampling rate and more taps require more CPU cycles for processing.

ST supplies the PDM2PCM software library to convert a PDM bit stream into a PCM audio stream [39,40] with the function of decimating and filtering out the PDM stream. It has a limited sampling rate (768 kHz–2.048 MHz) and a PCM output stream with 16-bit resolution. The purpose of this library focuses on basic audio applications with lower frequencies.

#### Acoustic Sensor Integration

**Figure 11:**
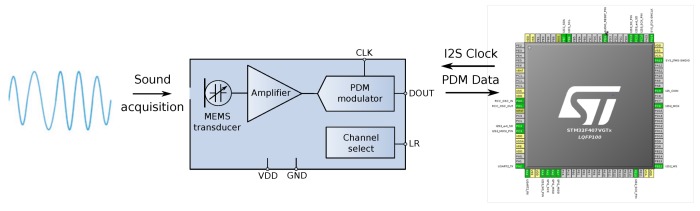
PDM digital microphone MP45DT02 block diagram and connection.

**Figure 12:**
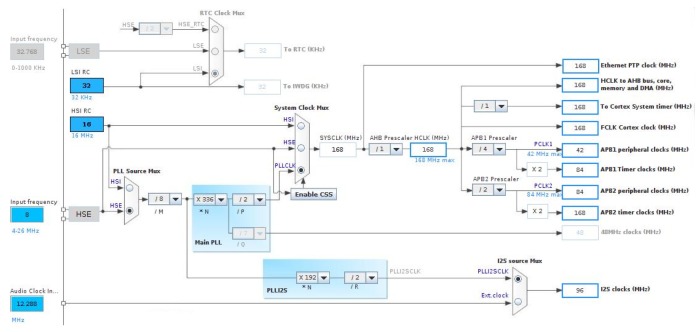
Clocks configuration in STM32F407VG SoC for PDM acquisition.

The main parts of a digital microphone are depicted in Figure 11. The Clock input (CLK) was used to control the PDM modulator. The range for the MP45DT02 mic goes from 1 MHz–3.25 MHz, and according to the desired sampling rate (48 kHz), it will be fixed to 3.072 MHz. This value has been calculated for a discrete-time representation (PDM bitstream) with an oversampling rate and a corresponding decimation factor of 64. To drive an I2S clock of such precise frequency, PLL clock resources are configured in the SoC from the 8 MHz HSE clock input to the PLL I2S clock by pre-scaling to 96 MHz and, next, by the I2S clock internal divisor to obtain the desired frequency, as shown in the bottom of Figure 12.

The MP45DT02 is designed to allow stereo audio capture. Two of the devices can operate simultaneously, sharing a bus. The microphone’s output is driven to the proper level on a selected clock edge and then goes into a high impedance state for the other half of the clock cycle (Figure 11 right). The channel select pin LR (Left-Right) defines the clock edge on which the digital microphone outputs valid data, and it is connected to GND for mono mode (“Left” channel) on the falling edge of the clock. The microphone will generate valid data for half of the clock period, then goes into a high impedance state for the other half.

The samples acquired by the SPI block, in I2S half-duplex master mode, were stored into a buffer memory using a DMA channel with interrupt signaling through the receive data register configured to provide 16 contiguous bits in MSB–first (left justified) format.

### 4.3. Position Decoding

**Figure 13:**
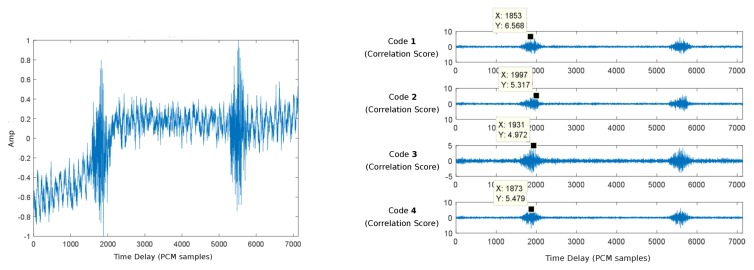
Relative positions extracted from the cross-correlation of the acquired signal with the four Kasami codes.

Figure 13 represents an acquired signal (left) and the relative positions extracted from the cross-correlation of this signal with the four Kasami codes assigned to the different beacons (right).

Following Algorithm 1, the received signal is correlated with the four Kasami codes associated with the different beacons, thus computing the DToAs that are later used by Algorithm 2 to determine the position of the receiver. This DToAs computation process is performed following an adaptive approach that started with the correlation of the sorter codes (63-bit). If the corresponding correlation peaks exceed a certain validation threshold, DToAs are computed and sent to the position determination algorithm. Otherwise, new correlations are subsequently performed with the longer 255- and 1023-bit sequences until valid correlation peaks are obtained.
**Algorithm 1:** PeaksDet
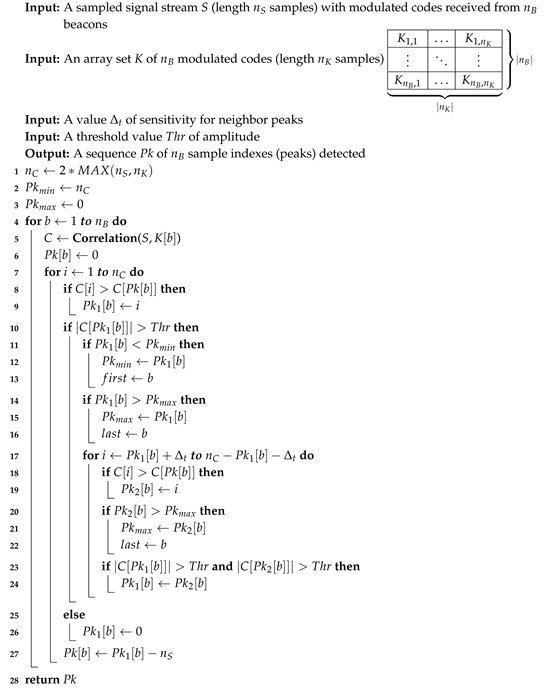

**Algorithm 2:** Positioning
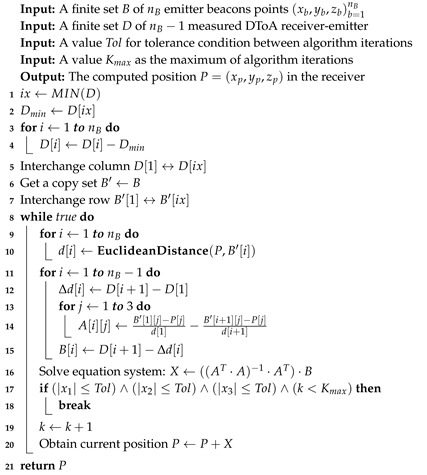


### 4.4. Calibration Process

The proposed procedure of self-calibration is a functionality introduced in this work with the purpose of easing the deployment of the beacons, without having to know previously their position, and taking advantage of the same digital processing of acoustic signals that are in the indicated positioning. To do this, with the same receiver, positioning measures were taken from a set of five points of known coordinates into the environment, and the combination of such measurements, with the procedure described below, and the implementation of the corresponding Algorithm 3, enabled obtaining from the its own receiver the reference coordinates of the beacons for the subsequent positioning of the same receiver within that environment.

Consider that there are *P* calibration points located at coordinates ${\left(x_{p},y_{p},z_{p}\right)}_{p=1}^{P}$ and *B* emitters with unknown coordinates ${\left(x_{b},y_{b},z_{b}\right)}_{b=1}^{B}$. Let $T_{p}$ be the time of flight from the source to the microphone at point *p*, and let *v* be the speed of sound in air according to measured temperature ϑ. Then, the distance between the source and point *p* can be written as:

$$\begin{array}{c} R_{p}=v_{air}\cdot T_{p}\\ v_{air}=331.3*\sqrt{1+\frac{\vartheta }{273.15}} \end{array}$$

and the difference in the time of flight between point *p* and Point 1 as:

(5) $$\tau_{p}=T_{p}-T_{1}$$

Multiplying Equation (5) by *v* yields:

$$\begin{array}{c} v\tau_{p}=vT_{p}-vT_{1}=R_{p}-R_{1} \end{array}$$

and therefore:

$$\begin{array}{c} 1-1R_{p}^{2}={\left(v\tau_{p}+R_{1}\right)}^{2}=v^{2}\tau_{p}^{2}+2v\tau_{p}R_{1}+R_{1}^{2} \end{array}$$

Rearranging some terms, we can write:

(6) $$0=v\tau_{p}+2R_{1}+\frac{R_{1}^{2}-R_{p}}{v\tau_{p}}\;\;\;\;\mathrm{for}\;\;p=2,3,\ldots ,P$$

Now, subtracting Equation (6) with p=2 from the same Equation with p=3,4,…,P, the following set of equations is obtained:

(7) $$0=v\tau_{p}-v\tau_{2}+\frac{R_{1}^{2}-R_{p}^{2}}{v\tau_{p}}-\frac{R_{1}^{2}-R_{2}^{2}}{v\tau_{2}}\;,\mathrm{for}\;\;p=3,4,\ldots ,P$$

Then, substituting the expressions:

$$\begin{array}{c} R_{p}=\sqrt{{\left(x_{p}-x\right)}^{2}+{\left(y_{p}-y\right)}^{2}+{\left(z_{p}-z\right)}^{2}}\\ \Rightarrow R_{p}^{2}=x_{p}^{2}-2x_{p}x+x^{2}+y_{p}^{2}-2y_{p}y+y^{2}+z_{p}^{2}-2z_{p}z+z^{2}\\ \Rightarrow R_{1}^{2}-R_{p}^{2}=x_{1}^{2}+y_{1}^{2}+z_{1}^{2}-x_{p}^{2}-y_{p}^{2}-z_{p}^{2}-2x_{1}x-xy_{1}y-2z_{1}z+2x_{p}x+2y_{p}y+2z_{p}z \end{array}$$

into Equation (7) yields:

(8) $$\begin{array}{cc} 0=v\tau_{p}-v\tau_{2} & +\frac{1}{v\tau_{p}}\left(x_{1}^{2}+y_{1}^{2}+z_{1}^{2}-x_{p}^{2}-y_{p}^{2}-z_{p}^{2}-2x_{1}x-xy_{1}y-2z_{1}z+2x_{p}x+2y_{p}y+2z_{p}z\right)\\ & -\frac{1}{v\tau_{p}}\left(x_{1}^{2}+y_{1}^{2}+z_{1}^{2}-x_{2}^{2}-y_{2}^{2}-z_{2}^{2}-2x_{1}x-2y_{1}y-2z_{1}z+2x_{2}x+2y_{2}y+2z_{2}z\right) \end{array}$$

Rewriting Equation (8) more succinctly, we have:

$$\begin{array}{c} 0=D_{p}+A_{p}x+B_{p}y+C_{p}z \end{array}$$

where:

$$\begin{array}{cc} A_{p} & =\frac{1}{v\tau_{p}}\left(-2x_{1}+2x_{p}\right)-\frac{1}{v\tau_{2}}\left(2x_{2}-2x_{1}\right)\\ B_{p} & =\frac{1}{v\tau_{p}}\left(-2y_{1}+2y_{p}\right)-\frac{1}{v\tau_{2}}\left(2y_{2}-2y_{1}\right)\\ C_{p} & =\frac{1}{v\tau_{p}}\left(-2z_{1}+2z_{p}\right)-\frac{1}{v\tau_{2}}\left(2z_{2}-2z_{1}\right) \end{array}$$

and:

(9) $$\begin{array}{c} D_{p}=v\tau_{p}-v\tau_{2}+\frac{1}{v\tau_{p}}\left(x_{1}^{2}+y_{1}^{2}+z_{1}^{2}-x_{p}^{2}-y_{p}^{2}-z_{p}^{2}\right)-\frac{1}{v\tau_{2}}\left(x_{1}^{2}+y_{1}^{2}+z_{1}^{2}-x_{2}^{2}-y_{2}^{2}-z_{2}^{2}\right) \end{array}$$

for p=3,4,…,P

The above set of P−2 expressions from Equation (9) can be written in matrix form as:

$$\left[\begin{array}{ccc} A_{3} & B_{3} & C_{3}\\ A_{4} & B_{4} & C_{4}\\ \vdots & \vdots & \vdots \\ A_{P} & B_{P} & C_{P} \end{array}\right]\left[\begin{array}{c} x\\ y\\ z \end{array}\right]=\left[\begin{array}{c} -D_{3}\\ -D_{4}\\ \vdots \\ -D_{P} \end{array}\right]$$

Applying a pseudo-inverse method to both sides of the matrix, it is possible to solve x,y,z for one beacon. Iteratively, for the *B* emitters in coordinates, $\left(x_{b},y_{b},z_{b}\right)$ for b=1,2,…,B can be obtained. Note that this algorithm yields a solution only when P≥5. In other words, five or more calibration points are needed. The iterative process to obtain the whole beacon set is described in Algorithm 3.
**Algorithm 3:** Calibration
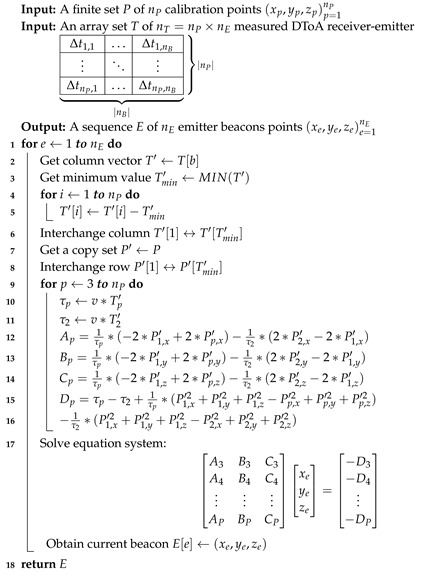


## 5. Experimental Results

In order to analyze the accuracy of the proposed positioning system, a 3D experimental environment has been configured, where the hardware of four wireless synchronized beacons, described in Section 3, and a portable wireless receiver (Section 4) have been deployed. The position values computed from the measurements taken by the receiver were sent to a mobile terminal, wirelessly connected with the receiver, as depicted in Figure 1.

**Figure 14:**
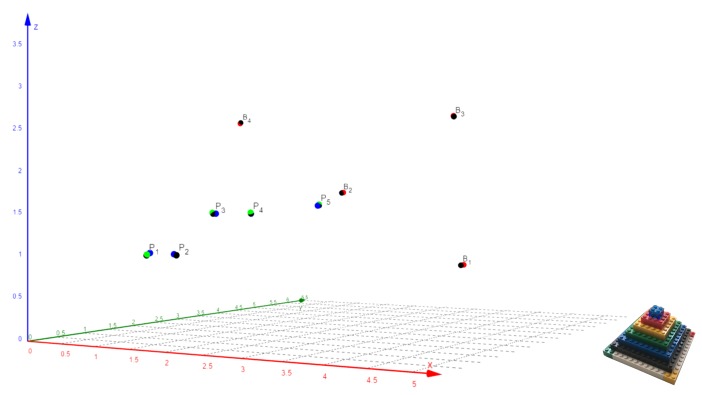
Experimental measures and calibration pyramid Lego.

A set of five test points $P_{i}$ have been defined, whose reference coordinates, as well as those of beacons $B_{j}$ have been accurately measured by using a precision laser instrumentation. All these coordinates are represented with black circles in Figure 14 (left) and are also included in the first three columns of Table 1, Table 2 and Table 3.

As explained before, in the encoded signal detection procedure, the beacons emitted the longest codes (1023 bit), and the receivers performed the initial correlation with the shortest codes (63 bit). If the amplitude threshold was not reached, they would perform a new attempt with longer codes (255 bit) and then a final attempt with the longest codes (1023 bit), following an adaptive approach.

**Figure 15:**
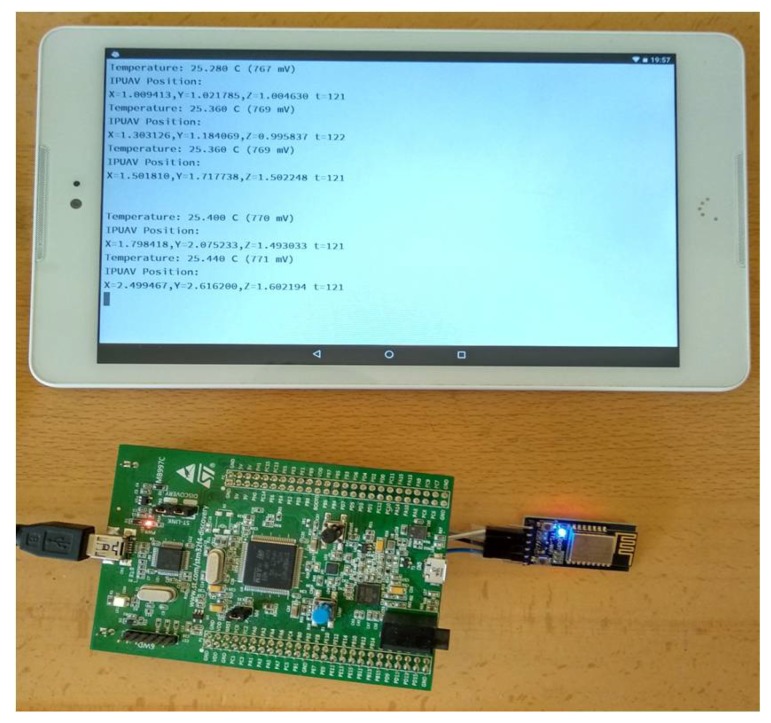
Picture of the acoustic receiver and the portable device where the positioning data are remotely acquired and shown.

Figure 15 depicts the remote acquisition in a portable device of the positioning data computed by the acoustic receiver. This portable device was connected with the receiver via a WiFi local network, and it showed textual information corresponding to the position of the MEMS microphone included in the receiver, as well as the ambient temperature measured by the sensor included in the SoC. This ambient temperature was used to estimate the speed of sound in air and, from it, the DToAs of the detected signals.

The first computed coordinates for the test points $P_{i}$ (green circles) were estimated from the ground-truth beacons coordinates introduced directly in the receiver. These values and derived statistics are shown in Table 1.

The following experimental coordinates calculated in the receiver are those estimated from the beacons calibration procedure. The Lego pyramid was placed with its blue vertex in $P_{1}$ and in parallel to the coordinates axes established in the room. Then, measurements were taken with the receiver from the rest of the vertices with a pre-established sequence and with the help of a push-button on the receiver board. These computed $B_{j}$ position coordinates (red circles), returned to the mobile terminal by the receiver, are shown in Table 2 together with their derived statistics.

Finally, new experimental measurements were taken based on those previously acquired in the calibration phase, already updated in the receiver, for the same $P_{i}$ test points (blue circles). The derived results are shown in Table 3 and serve to conduct a comparison of errors that is also reflected in the conclusions of Section 6.

In all results’ tables, the errors in the three coordinates are shown in the fourth column group, and the 3D positioning Percent Relative Error (PRE) and Mean Squared Error (MSE) are shown in the two last ones. It is worth mentioning that the errors made in the beacons calibration process (Table 2) are slightly below those made in the test points positioning (Table 1), a fact that can be attributed to the use of five reference calibration points in the latter case, with respect to the four used in the estimation of the test points’ positions.

The final Table 3 shows the measurement errors obtained during normal operation of the receiver, once the initial calibration process has been conducted in the currently-covered 3D environment. Note that these errors are greater than those appearing in the previous tables, mainly due to an accumulation of them, although their value is still around several centimeters, a result that can be considered acceptable for the applications for which the system is intended.

## 6. Conclusions

This works has presented the hardware/software co-designed infrastructure of an acoustic local positioning system, based on a set of wireless synchronized active beacons that cover an indoor environment. These beacons emit spread-spectrum signals (BPSK modulated Kasami codes) to a set of mobile receivers with computing capabilities to first estimate the beacons’ position by means of an auto-calibration process and, then, compute its own position by following a 3D multilateration procedure.

Both types of system nodes are based on integrated SoC platforms, with omni-directional digital MEMS microphones in the case of the receivers and advanced audio DACs and RF communication modules coupled. Furthermore, the implementation of algorithms and digital signal processing methods, optimized for high performance and low-power consumption, have been presented. A set of experimental results has been conducted to evaluate the accuracy of the proposed system, obtaining 3D-positioning mean squared errors between 2.2 and 10.2 cm when estimating the receiver’s location from the beacons’ calibrated coordinates.

As near future improvements, we intend to combine the inertial MEMS sensors available in the receiver platform, namely the gyroscope and the accelerometer, in order to complement the position estimation algorithm; as well as to initiate the transition of the receiver’s implementation to a low consumption platform, with similar performance to that used for the emitters.

## Figures and Tables

**Table 1:** Experimental measurements of test positions. PRE, Percent Relative Error.

$P_{i}$	Coordinates (m)			Measures (m)			Absolute Error (m)			PRE	MSE (m)
	Axis x	Axis y	Axis z	Axis x	Axis y	Axis z	Axis x	Axis y	Axis z		
$P_{1}$	1	1	1	1.0101	0.9931	1.0098	−0.0101	0.0069	−0.0098	2.67%	0.0157
$P_{2}$	1.3	1.2	1	1.2907	1.2092	1.0007	0.0093	−0.0092	−0.0007	1.55%	0.0131
$P_{3}$	1.5	1.7	1.5	1.4909	1.6988	1.5107	0.0091	0.0012	−0.0107	1.39%	0.0141
$P_{4}$	1.8	2.1	1.5	1.8007	2.0949	1.5096	−0.0007	0.0051	−0.0096	0.92%	0.0109
$P_{5}$	2.5	2.6	1.6	2.4994	2.6031	1.6107	0.0006	−0.0031	−0.0107	0.81%	0.0112

**Table 2:** Experimental measurements in the calibration process.

$B_{j}$	Coordinates (m)			Measures (m)			Absolute Error (m)			PRE	MSE (m)
	Axis x	Axis y	Axis z	Axis x	Axis y	Axis z	Axis x	Axis y	Axis z		
$B_{1}$	3.376	5.109	0.743	3.3998	5.1509	0.7485	0.0238	0.0419	0.0055	2.25%	0.0162
$B_{2}$	1.785	4.553	1.755	1.8004	4.5702	1.7600	0.0154	0.0172	0.0050	1.52%	0.0079
$B_{3}$	3.478	4.619	2.795	3.4671	4.6316	2.8058	−0.0109	0.0126	0.0108	0.97%	0.0066
$B_{4}$	0.055	4.581	2.803	0.0444	4.5816	2.7917	−0.0106	0.0006	−0.0113	24.34%	0.0052

**Table 3:** Experimental measures of positions with calibrated beacons.

$P_{i}$	Coordinates (m)			Measures (m)			Absolute Error (m)			PRE	MSE (m)
	Axis x	Axis y	Axis z	Axis x	Axis y	Axis z	Axis x	Axis y	Axis z		
$P_{1}$	1	1	1	1.0022	1.0639	1.0254	0.0022	0.0639	0.0254	8.70%	0.0688
$P_{2}$	1.3	1.2	1	1.2645	1.1986	1.0121	−0.0355	−0.0014	0.0121	4.12%	0.0375
$P_{3}$	1.5	1.7	1.5	1.5237	1.7268	1.4993	0.0237	0.0268	−0.0007	3.15%	0.0358
$P_{4}$	1.8	2.1	1.5	1.7487	2.1876	1.4939	−0.0513	0.0876	−0.0061	7.35%	0.1017
$P_{5}$	2.5	2.6	1.6	2.4964	2.5784	1.5962	−0.0036	−0.0216	−0.0038	1.22%	0.0222

## Abbreviations

The following abbreviations are used in this manuscript:

ALPS	Acoustic Local Positioning Systems
SoC	System on a Chip
RF	Radio Frequency
RT	Real-Time
BSP	Board Support Package
MEMS	MicroElectroMechanical Systems
HW	Hardware
SW	Software
FW	Firmware
TOA	Time of Arrival
TDOA	Time Difference of Arrival
TDMA	Time Division Multiple Access
CDMA	Code Division Multiple Access
FSK	Frequency Shift Keying modulation
OOK	On-Off Keying modulation
RSS	Received Signal Strength
SNR	Signal-to-Noise Ratio
PN	Pseudo-Noise code
dBFS	deciBels Full Scale
dBSPL	deciBels Sound Presure Level
AOP	Acoustic Overload Point
ARM	Advanced RISC Machine, ARM Holdings
STM32	STMicroelectronics 32-bit
DSP	Digital Signal Processing
FPU	Floating Point Unit
ART	Adaptive Real-Time accelerator
PDM	Pulse-Density Modulation
PCM	Pulse-Code Modulation
OSR	Over-Sampling Rate
HLGA	Holed Land Grid Array device package
LSB	Least Significant (right-most) Bit
MSB	Most Significant (left-most) Bit
LPF	Low-Pass Filter
HPF	High-Pass Filter
BPF	Band-Pass Filter
FIR	Finite Impulse Response filter
MA	Moving Average
SPI	Serial Peripheral Interface
IC	Integrated Circuit
I2S	Inter-IC Sound audio protocol
PLL	Phase-Looked Loop
SAI	Serial Audio Interface
UAV	Unmanned Aerial Vehicle
